# Listeria monocytogenes Source Distribution Analysis Indicates Regional Heterogeneity and Ecological Niche Preference among Serotype 4b Clones

**DOI:** 10.1128/mBio.00396-18

**Published:** 2018-04-17

**Authors:** Sangmi Lee, Yi Chen, Lisa Gorski, Todd J. Ward, Jason Osborne, Sophia Kathariou

**Affiliations:** a Department of Food, Bioprocessing and Nutrition Sciences, North Carolina State University, Raleigh, North Carolina, USA; b Department of Statistics, North Carolina State University, Raleigh, North Carolina, USA; c Division of Microbiology, Center for Food Safety and Applied Nutrition, Food and Drug Administration, College Park, Maryland, USA; d Produce Safety and Microbiology Research Unit, Agricultural Research Service, U.S. Department of Agriculture, Albany, California, USA; e Agricultural Research Service, U.S. Department of Agriculture, Peoria, Illinois, USA; University of Illinois at Chicago

**Keywords:** *Listeria monocytogenes*, source distribution, emerging clones, serotype 4b

## Abstract

Biodiversity analysis of the foodborne pathogen Listeria monocytogenes recently revealed four serotype 4b major hypervirulent clonal complexes (CCs), i.e., CC1, CC2, CC4, and CC6. Hypervirulence was indicated by overrepresentation of these clones, and serotype 4b as a whole, among human clinical isolates in comparison to food. However, data on potential source-dependent partitioning among serotype 4b clones in diverse regions are sparse. We analyzed a panel of 347 serotype 4b isolates, primarily from North America, to determine the distribution of clones in humans, other animals, food, and water. CC1, CC2, CC4, and CC6 predominated, but surprisingly, only three clones, i.e., CC2 and the singleton sequence types (STs) ST382 and ST639, exhibited significant source-dependent associations, with higher propensity for food (CC2) or water (ST382 and ST639) than other sources. Pairwise comparisons between human and food isolates identified CC4 as the only serotype 4b clone significantly overrepresented among human isolates. Our analysis also revealed several serotype 4b clones emerging in North America. Two such emerging clones, ST382 (implicated in several outbreaks since 2014) and ST639, were primarily encountered among human and water isolates. Findings suggest that in spite of the ubiquity of CC1, CC2, CC4, and CC6, regional heterogeneity in serotype 4b is substantially larger than previously surmised. Analysis of even large strain panels from one region may not adequately predict clones unique to, and emerging in, other areas. Serotype 4b clonal complexes may differ in ecological niche preference, suggesting the need to further elucidate reservoirs and vehicles, especially for emerging clones.

## IMPORTANCE

In Listeria monocytogenes, serotype 4b strains are leading contributors to human disease, but intraserotype distributions among different sources and regions remain poorly elucidated. Analysis of 347 serotype 4b isolates from four different sources, mostly from North America, confirmed the overall predominance of the major clones CC1, CC2, CC4, and CC6 but found that only CC4 was significantly associated with human disease, while CC2 was significantly associated with food. Remarkably, several emerging clones were identified among human isolates from North America, with some of these also exhibiting a propensity for surface water. The latter included the singleton clones ST382, implicated in several outbreaks in the United States since 2014, and ST639. These clones were noticeably underrepresented among much larger panels from other regions. Though associated with North America for the time being, they may eventually become globally disseminated through the food trade or other venues.

## INTRODUCTION

Listeria monocytogenes is a bacterial foodborne pathogen that can cause severe disease (listeriosis) in humans and other animals. Human listeriosis has high hospitalization and case fatality rates with symptoms that include septicemia, meningitis, stillbirths, and abortions (1–3). Contamination of food by L. monocytogenes typically involves transfer of the pathogen from food-processing environments and equipment which can become persistently contaminated (4–6). Persistence in food-processing facilities is mediated by several attributes of L. monocytogenes, including biofilm formation, tolerance to cold, and resistance to disinfectant and phage (4–7).

The 13 identified *L. monocytogenes* serotypes are grouped into four genetic lineages. Three serotypes, specifically 1/2a (lineage II), 1/2b, and 4b (both lineage I, although some 4b strains are lineage III or IV), are implicated in over 95% of the human clinical cases (4, 8). Serotype 4b strains within lineage I, in particular, exhibit the strongest epidemiological association with human listeriosis (9–11). In comparison to the other serotypes, 4b is more likely to be overrepresented among human clinical isolates than those from food (9–13). In agreement with such distributions, whole-genome sequence (WGS)-based analysis revealed that genes mediating biosynthesis of serotype 4b-specific teichoic acid (14, 15) were among the three leading categories of genetic determinants overrepresented in isolates from human listeriosis in comparison to those from food; the other two were full-length internalin A (InlA) and *Listeria* pathogenicity island 3 (LIPI-3), which harbors the listeriolysin S gene cluster (9).

L. monocytogenes strains are delineated into sequence types (STs) based on conventional multilocus sequence typing (MLST) which utilizes seven alleles. STs are then grouped into clonal complexes (CCs) with strains in the same CC sharing at least six of the seven MLST alleles. Within serotype 4b, CC1, CC2, and CC6, which were previously designated epidemic clone I (ECI), ECIa/IV, and ECII, respectively, and the more recently recognized CC4 represent large, widely disseminated clones that make major contributions to human listeriosis (9, 16–22).

In a recent landmark study, Maury et al. (9) described that, compared across the entire L. monocytogenes species, CC1, CC2, CC4, and CC6 were significantly more common among clinical isolates than might be expected based on their prevalence in food. These clones are considered “hypervirulent” based on their relative incidence in disease versus food, propensity to cause illness in individuals with relatively few comorbidities, and capacity to breach blood-brain and blood-placenta barriers (9). Overrepresentation in human isolates in comparison to food was especially noteworthy for CC4, for which an estimated 71.3% of the isolates were of human clinical origin (9). However, serotype 4b clonal partitioning among different sources remains poorly elucidated. In addition, the study by Maury et al. focused exclusively on isolates from France from 2005 to 2013 (9). There is a clear need for analysis of the distribution of different serotype 4b clones across different sources from other regions. In this study, we analyzed source-associated distributions of L. monocytogenes serotype 4b mostly from North America and from four sources including human listeriosis, nonhuman animals, food/food processing facilities, and the natural environment, primarily surface water.

## RESULTS AND DISCUSSION

Of the 347 serotype 4b isolates, most (329, 94.8%) were lineage I; 17 (4.9%) were lineage III, while lineage IV was detected only once (see Tables S1 and S2 in the supplemental material). The infrequent presence of serotype 4b strains in lineage III, which primarily consists of isolates of serotypes 4a and 4c, has been documented (23, 24). Use of the multiplex PCR scheme yielded the typical serotype 4b PCR profile for all isolates except those of ST218, ST382, and CC554, which exhibited the IVb-v1 profile reported previously for these clones (22). The multilocus genotyping (MLGT) scheme (25, 26) and WGS data for selected isolates failed to reveal premature stop codons in *inlA* among any of the 347 isolates, suggesting that they harbored full-length *inlA*, regardless of source or lineage. The tendency of serotype 4b to harbor full-length InlA has been documented (9, 12, 13, 27, 28).

10.1128/mBio.00396-18.1 TABLE S1  List of serotype 4b Listeria monocytogenes isolates investigated in this study. Download TABLE S1, DOCX file, 0.04 MB. Copyright © 2018 Lee et al. 2018 Lee et al. This content is distributed under the terms of the Creative Commons Attribution 4.0 International license.

10.1128/mBio.00396-18.2 TABLE S2  Statistical analysis of the source distribution of serotype 4b Listeria monocytogenes clones. Download TABLE S2, DOCX file, 0.02 MB. Copyright © 2018 Lee et al. 2018 Lee et al. This content is distributed under the terms of the Creative Commons Attribution 4.0 International license.

**FIG 1:**
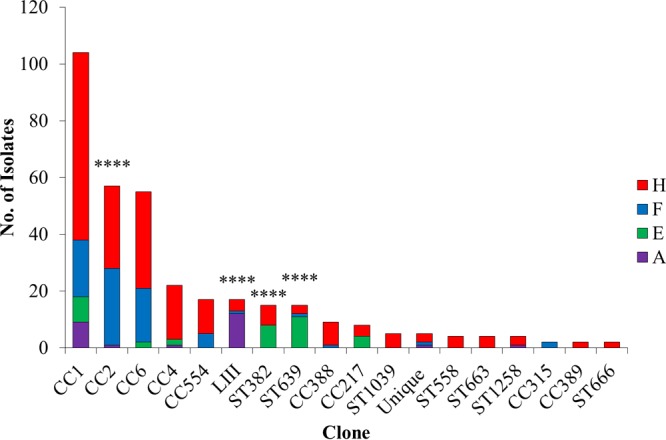
L. monocytogenes clones in the serotype 4b strain panel. Relative abundance of each clone in the panel is shown in decreasing order. CC indicates clonal complex, and singletons are indicated with the corresponding sequence type (ST). “Unique” is a group that consists of four lineage I clones (CC218, ST688, ST1061, and ST1256) encountered only once in the panel and the lineage IV clone ST563 (Table S2). “LIII” consists of the 17 lineage III isolates (Tables S1 and S2). Color codes for each source are shown at right (red, human; blue, food; green, environment; purple, animal). Source designations are as described in Materials and Methods and in Tables S1 and S2. Significantly different distributions of specific clones across all sources are indicated with **** (*P* < 0.0001).

**FIG 2:**
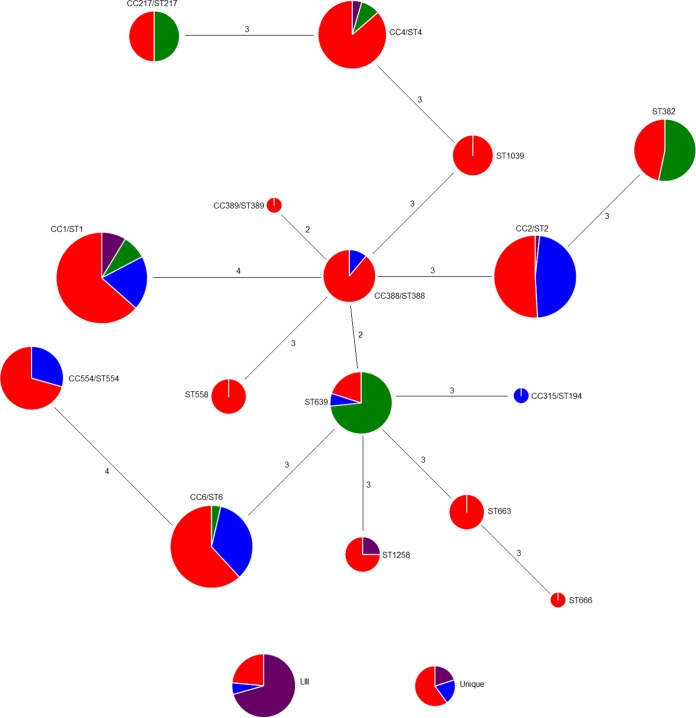
MST of the lineage I serotype 4b isolates. An MST was constructed for the 16 lineage I clones that were encountered among ≥2 isolates. A commonly encountered ST of each CC was arbitrarily chosen to construct the MST. Lineage III clones are included at the bottom of the figure (“LIII”) but are not part of the MST. Unique lineage I clones (encountered only once) and the sole lineage IV clone are also included (“Unique”) at the bottom of the figure but are not part of the MST. Each clonal group is illustrated as a pie chart, with different colors representing different sources (red, human; blue, food; green, environment; purple, animal). The CC and ST designations are included near each pie chart. The size of each circle is proportional to the log value of the number of strains in each clone. Numbers on the connecting lines represent the number of allelic differences between clones.

Of the 34 clones detected in the strain panel, four lineage I clones were the most common. These were CC1 (formerly designated epidemic clone I [ECI]), followed by CC2 and CC6 (formerly designated ECIa/ECIV and ECII, respectively) and the newly described clone CC4 (9) (Fig. 1; Table S2). The predominance of CC1, CC2, and CC6 among serotype 4b isolates of L. monocytogenes has been repeatedly demonstrated (9, 16, 17, 20–22). Multiple other clones were repeatedly encountered in the strain panel, and the diversity within the lineage I strain panel is illustrated with a minimum spanning tree (MST) (Fig. 2). It was noteworthy that several clones that were detected relatively frequently (>4%), e.g., ST382 and CC554 (both with the IVb-v1 multiplex PCR profile) and ST639, were absent from previous studies that surveyed large strain panels of L. monocytogenes (17); the attributes of these clones will be discussed further below. Altogether, the panel consisted of serotype 4b isolates representing 20 clones of lineage I, 13 of lineage III, and one of lineage IV. In summary, our serotype 4b strain panel reflects the diversity of the serotype 4b L. monocytogenes population, including not only the well-known major clones but also those exhibiting the IVb-v1 molecular serotype profile and clones from lineages III and IV.

### Source-associated partitioning is uncommon in serotype 4b L. monocytogenes.

Isolates of the same clone tended to be derived from multiple sources among the four that were considered, i.e., “human” (human clinical isolates), “food” (food/food processing facilities), “animal” (nonhuman animals), and “environment” (natural environment, mostly water) (Fig. 1). Exceptions in lineage I were a few relatively uncommon clones, such as ST1039, ST558, ST663, CC389, and ST666, which were only of human origin, and CC315 (food) (Fig. 1 and 2). The small number of isolates in these clones may account for their absence in other sources. With the exception of CC315, which was previously detected frequently from animals and humans though uncommonly from food, these clones were absent in previous surveys (9, 17).

**FIG 3:**
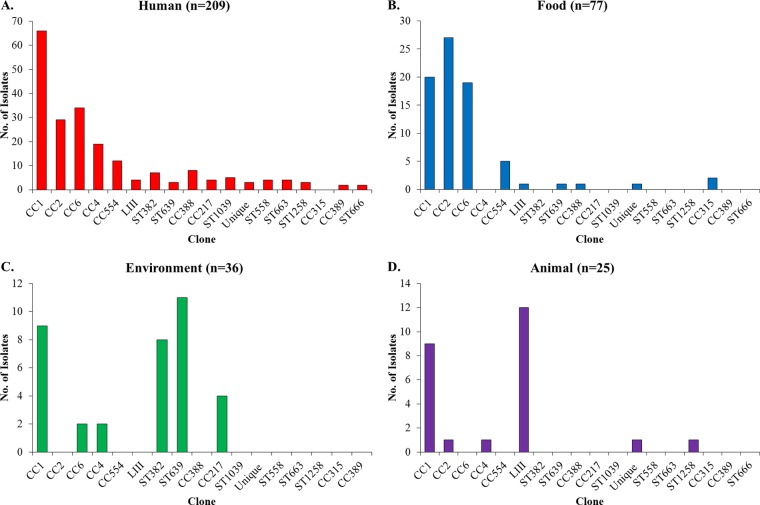
Numerical representation of clones from each source. Sources are human (A), food (B), environment (C), and animals (D). Source designations, CC and ST designations, and the group designations “Unique” and “LIII” are as in Fig. 1.

When the different sources were examined separately, all but one of the 20 lineage I clones (the exception being CC315) were encountered among human isolates, albeit with varying frequency, with CC1, CC2, CC4, and CC6 being the most predominant (Fig. 3A). In contrast, 11 of the 20 lineage I clones were not detected among food isolates, which tended to be dominated by CC1, CC2, CC6, and CC554 (Fig. 3B). Environmental isolates were represented by only 6 clones, primarily CC1, ST382, ST639, and CC217 (Fig. 3C). Last, among animal isolates CC1 was the only lineage I clone with important contributions; as discussed above, animal isolates also had significant representation of lineage III (Fig. 3D). Our findings contrast somewhat with those from a previous analysis with mostly European isolates, which indicated not only CC1 but also CC2, CC4, and CC315 as predominant serotype 4b contributors to animal isolates of lineage I (17).

Statistical analysis of source distributions of the different clones revealed that significant source associations were in fact uncommon: only CC2, ST382, ST639, and lineage III (considered a single group of all lineage III isolates) exhibited significantly different relative abundance across the four sources (Fig. 1). Specifically, CC2 was significantly more common among food isolates than among those of human, animal, or environmental origin (Table S2). Two other clones, the singletons ST382 and ST639, were significantly more common among isolates from the environment (water) than those of human, food, or animals. Last, lineage III was significantly more common among isolates from animals than those of human, food, or environmental origin (Table S2), supporting the previously documented association of this group with nonhuman animals (12). However, and as will be discussed further below, even though most lineage III isolates were of animal origin, lineage I (mostly CC1) still accounted for a substantial fraction of the animal-derived serotype 4b isolates in our panel (Fig. 1; Table S2). Our findings that most clonal groups of serotype 4b L. monocytogenes were found in multiple sources without particular source bias support previous conclusions (17) and make the observed source association of CC2, ST382, and ST639 especially noteworthy.

### Of the previously reported hypervirulent clones, only CC4 is overrepresented among human clinical isolates.

**FIG 4:**
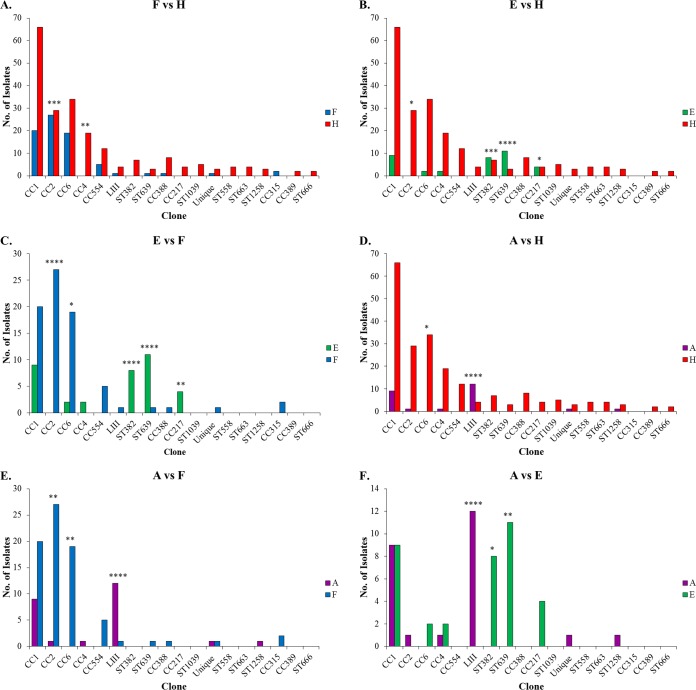
Numerical representation of each clone in pairwise comparisons between sources. Source comparisons are food versus human (A), environment versus human (B), environment versus food (C), animal versus human (D), animal versus food (E), and animal versus environment (F). Source designations, CC and ST designations, and the group designations “Unique” and “LIII” are as in Fig. 1. Significantly different distributions between sources are indicated with *, **, ***, and **** (*P* < 0.05, 0.01, 0.001, and 0.0001, respectively).

As indicated above, none of the 18 clones that were encountered among >0.5% of isolates in our panel were found overrepresented among human isolates in comparisons involving all other sources (food, animals, and environment) (Table S2). However, pairwise comparisons identified certain clones that were overrepresented in one source versus another. Notably, only one clone, CC4, was found overrepresented among human isolates in comparison to those from food (Fig. 4A). On the other hand, pairwise comparisons affirmed the significant associations of CC2 with food, lineage III with animals, and ST382 as well as ST639 with environment (water) (Fig. 4; Table S2). In addition, in pairwise comparisons CC6 was significantly more common in food than in animals or the environment, while CC217 was overrepresented in the environment in comparison to its frequency among food or human isolates (Fig. 4; Table S2).

Remarkably, CC1, the most widely encountered clone, was not found to be significantly overrepresented among human isolates. Even though CC1 made the highest contribution to human isolates (Fig. 3A), with almost a third of all human isolates belonging to this clone, it also made major contributions to isolates from food as well as animals and the environment (Fig. 3B to D). In fact, as indicated above, CC1 was a leading contributor to animal isolates, second only to lineage III (Fig. 3D). Interestingly, the other three major clones (CC2, CC4, and CC6) were rare among the animal isolates (Fig. 3D; Table S2). CC1 prevalence among human isolates was not significantly higher than predicted based on its prevalence in food (*P* > 0.05). Similar findings were obtained with CC2 and CC6. After CC1, these two clones were the largest contributors to the human isolates (Fig. 3A) but were not significantly overrepresented among human isolates in comparison to their frequency in food (Table S2). In fact, as discussed above, CC2 was significantly overrepresented among food isolates in comparison to its frequency in human isolates (Fig. 4A; Table S2) and was the leading clone among food-derived isolates (Fig. 3B).

Our findings with CC4 are in agreement with a previous report by Maury et al. which examined a large panel of isolates of diverse serotypes of human and food origin in France and demonstrated that this clone was significantly more common among human isolates than those from food (9). However, in contrast to our findings, this previous study also reported a significantly higher association of CC1, CC2, and CC6 with human clinical origin than with food origin (9). The discrepancy is especially pronounced with CC2, which actually showed the reverse trend in our panel. The reasons for these differences may reflect the composition of the strain panels. Most of our human and food isolates were from North America, while the panel investigated by Maury et al. consisted exclusively of isolates from France (9). Factors that may contribute to regional differences in relative prevalence of certain strains in foods or clinical samples remain poorly understood but may include differences in the frequency of consumption of certain food commodities that may be associated with specific subtypes of L. monocytogenes. In addition, and of even more importance, our panel consisted of isolates that were exclusively of serotype 4b, while the panel investigated by Maury et al. included diverse serotypes (9). Certain lineage II clones in that panel, e.g., CC121 and CC9, of serotype 1/2a and 1/2c, respectively, were highly abundant among food isolates while underrepresented among those of human clinical origin (9). Significant overrepresentation among human isolates was noted for CC2 and CC6 only in comparisons across the whole species and not in comparisons across the lineage of these clones, i.e., lineage I, which includes serotypes 4b and 1/2b (9). Our examination of the serotype 4b population from the work of Maury et al. for which clone information was available (9) indicated that, when analyzed within the serotype 4b context, only CC1 and CC4 were significantly represented more highly among human isolates than predicted based on their prevalence in food (*P* < 0.01 and *P* < 0.0001, respectively). In contrast, CC6 was similarly represented among food and human isolates, while CC2 exhibited the same strong association with food origin that we observed in our panel (*P* < 0.0001).

In summary, our findings suggest that when examined specifically within the serotype 4b context, only one major clone, CC4, is significantly associated with human origin, while another major clone, CC2, is less commonly encountered among human isolates than might be predicted based on its high prevalence in food. Such findings reinforce the need for further studies to elucidate potentially unique adaptive attributes of these major clones that may predispose them to contaminate food with less-than-expected incidence in illness (CC2) or to cause human disease with less-than-expected incidence in food (CC4).

### Several serotype 4b clones appear to be emerging in North America, with three showing propensity for water.

It was remarkable that, with the exception of the four major clones CC1, CC2, CC4, and CC6, the other 12 lineage I clones constituting >0.5% of our isolates (Table S2) were largely unique to our panel. For instance, of these 12 clones only CC388 was detected among >0.5% of 2,197 serotype 4b isolates from France from 2005 to 2013 (9). Similar findings were obtained with another large study of approximately 2,000 isolates from diverse regions, with a majority from Europe (17). Besides CC1, CC2, CC4, and CC6, only CC315 and CC217 were in common with the lineage I clones constituting >0.5% of our panel (17). It is noteworthy that in that study all four isolates of CC217 were from North America with three from the natural environment (2002 to 2003) and one of human origin (17).

Interestingly, recent whole-genome-based subtyping by Moura et al. (22) revealed several of the clones appearing unique to our panel among isolates from North America, primarily the United States (22). Specifically, CC217, CC554, ST382, ST388, ST558, ST639, and ST663 were derived primarily from human isolates subsequent to 2013 (22). On the other hand, similar whole-genome-based subtyping of recent (2015 to 2016) human clinical isolates from France failed to identify these clones (29).

Several of these clones have been implicated in human listeriosis outbreaks in the United States. ST558 was first detected in an earlier (2000) outbreak of listeriosis in North Carolina, USA (30). Since 2014, ST382 has been implicated in three different multistate outbreaks of listeriosis in the United States, traced to fresh fruit and vegetables (31). Phylogenetic analysis suggests that this singleton clone is novel, having emerged from its closest ancestor approximately 32 years prior to 2016 (31). CC554 was also implicated in a recent produce-associated outbreak in the United States (32). ST382 and CC554 are among serotype 4b clones yielding a variant pattern (IVb-v1) by multiplex PCR but representing divergent clonal groups (22).

Of these clones, only CC554 was detected among multiple food isolates (Fig. 1; Table S2). Surprisingly, almost all isolates of ST382, ST639, and CC217 were derived from human listeriosis and the environment (watersheds), while the less-abundant clones ST558, ST663, ST666, and ST1039 were detected only among human isolates (Fig. 1; Table S2). It will be of interest to determine whether further surveillance may support a propensity of these clones for human disease, potentially reflecting hypervirulence attributes as observed for CC4 (9).

Unexpected findings were obtained regarding clonal partitioning among isolates from the natural environment (primarily water). CC2 and CC6 were significantly underrepresented in water in comparison to food, while three clones, i.e., CC217 and the singletons ST382 and ST639, were associated with watershed origin (Fig. 4C). Previous surveys of L. monocytogenes isolates from natural environments suggested higher prevalences of certain serotype 4b strains based on *sigB* subtyping or pulsed-field gel electrophoresis profiles, but the corresponding CC or ST designations were not provided (33, 34).

Mechanisms that may mediate possible amplification and persistence of CC217, ST382, and ST639 in the natural environment remain to be identified. Isolates with these STs were from watersheds in California (35). Indeed, the majority of L. monocytogenes isolates from a West Coast (California, USA) watershed survey were serotype 4b (35), and ST382, ST639, and CC217 were each detected in multiple watersheds (L. Gorski, unpublished findings). It will be of interest to determine distributions of these and other clones among serotype 4b L. monocytogenes isolates from surface waters in other regions. This will elucidate the extent to which some of these clones may be geographically restricted. Comparisons of our findings with those of other studies (e.g., references 33, 34, 36, and 37) are currently hampered by the lack of common genotyping tools but will be greatly facilitated by the employment of subtyping tools such as MLST, with portable, unambiguous outputs. This would allow much-needed metadata analyses to identify potential sources in the natural environment for strains contaminating food facilities and the food supply.

In the case of ST382, two of the three investigated outbreaks involved fresh produce on the West Coast (38, 39), and a West Coast origin for this clone was recently proposed (40). However, sporadic clinical isolates of ST382 in our panel originated from diverse regions in the United States (northeastern, southeastern, and midwestern states) (Table S1), supporting other studies of the wider distribution of this clone (31). Human CC217 and ST639 isolates were also derived from different regions in the United States (Table S1). Even though outbreak investigations involving CC217 and ST639 are not yet reported in the literature, their repeated identification among human isolates in this panel and in the WGS-based analysis of recent U.S. isolates from human listeriosis (22) clearly suggests pathogenic potential. It is noteworthy in this context that CC217, ST382, and ST639 all harbor the newly described *Listeria* pathogenicity island 4 (LIPI-4) (22), originally considered unique to CC4 and implicated in that clone’s enhanced neurovirulence and capacity for placental infection (9). PCR examination of our CC217, ST382, and ST639 isolates indeed confirmed the presence of LIPI-4 in both human and water-derived isolates (C. Parsons and S. Kathariou, unpublished data). The extent to which surface water may serve as a preferential habitat or reservoir for these clones remains to be elucidated.

To summarize, our findings provide evidence for clones currently emerging in North America, suggesting regional differences in the population structure of serotype 4b L. monocytogenes. Recent involvement of some of these clones in outbreaks renders them of special public health interest, and the apparent proclivity of some of these clones for the natural environment (water) reinforces the need to further explore surface water as a reservoir for L. monocytogenes.

### ST1214: human-adapted lineage III serotype 4b clone in the United States?

Five of the 209 human serotype 4b isolates in our panel were of lineage III, and three of these were ST1214 (Table S2). As previously hypothesized (20), lineage III ST1214 strains (MLGT haplotype Lm3.42) may have a propensity for human listeriosis, possibly through higher virulence. Earlier MLGT-based analysis of 90 lineage III isolates revealed that most (14/15) haplotype Lm3.42 isolates were from human listeriosis, in contrast to other lineage III haplotypes that were primarily found in animals (T. J. Ward, unpublished data).

### Conclusions.

Our analysis of L. monocytogenes source distribution indicates surprising regional heterogeneity and ecological niche preference among serotype 4b clones. The previously described hypervirulent, ubiquitous clones CC1, CC2, CC4, and CC6 were also the leading overall contributors to our panel. However, compared specifically in the context of serotype 4b, only CC4 was overrepresented among human isolates, supporting the recently described hypervirulence attributes of this clone (9). Frequencies of CC1 and CC6 among human isolates did not differ from what would be predicted based on incidence in food, while CC2 was overrepresented among food isolates. Such findings suggest that among serotype 4b isolates, the high incidence of CC1, CC2, and CC6 in disease may reflect frequent occurrence of these clones in food and raise the possibility that CC2 may not be as virulent as the other predominant clones.

We consider it remarkable that, besides the four major ubiquitous clones (CC1, CC2, CC4, and CC6), our panel included multiple additional clones that were not encountered before in much larger panels of isolates largely from France or elsewhere in Europe (9, 17, 29). Some of these clones have been recently involved in high-impact outbreaks in North America. Such data suggest that these represent emerging pathogenic L. monocytogenes clones in North America, and in fact evolutionary analysis provided strong evidence that one of these, ST382, emerged only about 30 years ago from its closest ancestor (31). Such findings suggest that serotype 4b may exhibit substantially more regional heterogeneity than previously surmised. Analysis of even large strain panels from one region may not adequately predict clones unique to, and emerging in, other areas. These findings highlight the continuing need to explore the biodiversity of L. monocytogenes for a richer, more complete picture of the distributions and source associations of distinct clonal groups. We expect that the North American emerging clones that we detected may eventually become disseminated to other continents through the global food trade or other venues. In fact, WGS analysis has already provided evidence suggesting that stone fruit implicated in an outbreak in the United States in 2014 (38) and exported to Australia was also implicated in a case of human listeriosis there (41).

Molecular mechanisms underlying the emergence of hypervirulent clones such as CC4 remain poorly understood. Interestingly, CC4 harbors all three known major pathogenicity islands of L. monocytogenes: *Listeria* pathogenicity island 1 (LIPI-1), LIPI-3, and LIPI-4 (9). LIPI-4, in particular, was associated with neurovirulence and placental infection in a murine model (9), and based on its sequence content, it appears to be acquired by horizontal gene transfer (HGT). Though first identified in CC4, it was later shown to be also harbored by some of the emerging clones in our panel (ST382, ST639, and CC217) (22). Clones such as CC4, CC217, ST382, and ST639 may thus be considered privileged in terms of their virulence repertoire, harboring all three major pathogenicity islands of L. monocytogenes: LIPI-1 (listeriolysin O [LLO] island), LIPI-3 (listeriolysin S island, also considered to be acquired by HGT), and LIPI-4 (neurovirulence/placental infection island). At this time, however, the global response of the genome to HGT-mediated acquisition of these islands remains poorly understood. Further experimental and epidemiological analyses are needed to characterize virulence of specific clones, as well as to elucidate their relative fitness in foods, food processing ecosystems, or the natural environment.

We found it of further interest that three of these emerging clones, specifically CC217 and the singletons ST382 and ST639, were significantly associated with watershed origin. This suggests the need to further investigate clonal prevalence in water and other environmental reservoirs, possibly yielding clues to the ultimate source of clones eventually contaminating food processing facilities and food. In contrast to the extensive investigations of population structure and adaptive attributes of L. monocytogenes from food processing environments, comparable understanding related to natural environments such as soil and water remains limited, clearly pointing to the need to further elucidate the nexus between natural ecosystems, food contamination, and disease.

## MATERIALS AND METHODS

### Bacterial strains and serotype determination.

A total of 347 serotype 4b L. monocytogenes isolates were examined in this study (see Table S1 in the supplemental material). Most of the isolates were from North America and are part of the L. monocytogenes strain collection at North Carolina State University. This serotype 4b panel included 209 isolates from human cases of listeriosis (designated “human”); 77 isolates from foods and food processing environments, collectively designated “food”; 36 from natural environments, primarily surface water (watersheds) and water treatment effluent, collectively designated “environment”; and 25 from nonhuman animals, collectively designated “animal” (Tables S1 and S2). The human panel included 136 previously described isolates from sporadic listeriosis in the United States from 2003 to 2008 (20). Food isolates included several that were previously described (42, 43) and were isolated by the Kathariou laboratory or by collaborators at the U.S. Food and Drug Administration; isolates from water treatment effluents were isolated by the Kathariou laboratory, while watershed isolates were from the Gorski laboratory and were isolated as described previously (35) (Table S1). To minimize redundancy, only one serotype 4b strain was included per positive food or environmental sample, and multiple isolates from known outbreaks were avoided. L. monocytogenes was cultured in brain heart infusion (BHI; Becton, Dickinson and Co., Sparks, MD) or on BHI plates containing 1.2% agar (Becton, Dickinson and Co.) at 37°C. Serotype was confirmed with the multiplex PCR-based serotyping scheme of Doumith et al. (44).

### Lineage determination, MLGT, and MLST-CC designations.

Lineage and MLGT haplotypes were determined as previously described (25, 26, 45). The MLGT scheme also yielded information on the presence of known premature stop codons of *inlA* (25). The MLST-based ST corresponding to each MLGT haplotype was identified via WGS analysis of a strain panel representing all known haplotypes (Y. Chen, T. Ward, and P. Evans, unpublished data); MLGT-MLST counterpart designations will be described in a separate publication. For isolates lacking MLGT haplotype designations, WGS analysis was employed to determine ST and CC. Novel STs were assigned following submission of sequence data to the *Listeria* MLST database (http://bigsdb.pasteur.fr). A clone corresponded to an MLST-based CC or, for singleton clones, to a ST as described previously (16). A MST was constructed using Ridom SeqSphere+ (Ridom GmbH, Muenster, Germany) for the 16 lineage I clonal groups, excluding clones that were represented by only one strain. We identified only the CC designation of each strain (Table S1), not the specific ST, and each CC could contain multiple STs. Therefore, to construct the MST, we arbitrarily chose one ST for each CC.

### Statistics.

To investigate association between clone and source frequencies, the Pearson χ^2^ statistic was computed from a two-way contingency table of 22 clones (row) and four sources (column), with lineage III isolates coalesced into a single group (corresponding to one of the 22 clones). Since the table was sparse with many zero cells and some small row totals, Monte Carlo estimates of exact *P* values for tests of association were computed with *n* = 100,000 simulations using the exact statement of the FREQ procedure in SAS (SAS Institute, Cary, NC). The observed value of the Pearson χ^2^ from the 22-by-4 table was 303.8 on 63 degrees of freedom. Both the Monte Carlo *P* value and the one from the reference χ^2^ distribution were <0.0001. To investigate the way in which the relative abundance of a clone may vary across sources, generalized linear models were used with column (source) totals as the size parameters (*n*) of assumed binomial distributions. In particular, the four totals were *n*_*A*_ = 25, *n*_*E*_ = 36, *n*_*F*_ = 77, and *n*_*H*_ = 209 for the animal, environment, food, and human isolates, respectively. The probability parameters (*P*) of these four binomial counts for each clone were modeled using factorial effects for source. For each clone, a test statistic was computed for the hypothesis of equal probabilities across the four sources. Last, all six pairwise comparisons of source-specific probabilities were carried out for each clone to further characterize abundance variation across sources. Fisher’s exact test was used for each such pairwise comparison. Fisher’s exact test was also used for analysis of human versus food distributions of 2,180 serotype 4b isolates for which clone information was available in the work of Maury et al. (9).
