# Supplementary material for: Listeria monocytogenes Source Distribution Analysis Indicates Regional Heterogeneity and Ecological Niche Preference among Serotype 4b Clones
Source: mBio. 2018 Apr 17;9(2):e00396-18. doi: 10.1128/mBio.00396-18 (PMC5904418; doi:10.1128/mBio.00396-18)
Supplement: TABLE S1 [file mbo002183818st1.docx]

| **Table S1. Serotype 4b *Listeria monocytogenes* isolates investigated in this study** | | | | | | |
| --- | --- | --- | --- | --- | --- | --- |
| **Isolate** | **Year^1^** | **State /Province, Country^1^** | **Source^2^** | **MLGT haplotype** | **Clone** | **Lineage^4^** |
| OLM 9 | 1933 | USA | A | 1.13_4b_Sw87_EC1 | CC1 | I |
| OLM 20 | 1937 | USA | A | 1.13_4b_Sw87_EC1 | CC1 | I |
| OLM 43 | 1949 | Ontario, Canada | A | 1.13_4b_Sw87_EC1 | CC1 | I |
| OLM 63 | 1951 | Japan | A | 1.13_4b_Sw87_EC1 | CC1 | I |
| OLM 65 | 1952 | Japan | A | 1.13_4b_Sw87_EC1 | CC1 | I |
| OLM 69 | 1952 | Israel | A | 1.13_4b_Sw87_EC1 | CC1 | I |
| OLM 66 | 1953 | Japan | A | 1.13_4b_Sw87_EC1 | CC1 | I |
| OLM 126 | 1959 | Canada | A | 1.13_4b_Sw87_EC1 | CC1 | I |
| SK3296 | 2013 | NC, USA | A | 1.13_4b_Sw87_EC1 | CC1 | I |
| OLM 127 | 1959 | Newfoundland, Canada | A | 1.2_4b_UK88_EC1a | CC2 | I |
| SK3564 | 2016 | NC, USA | A | ND^3^ | CC4 | I |
| OLM 39 | 1939 | IA, USA | A | Lm3.1 | ST261 | III |
| NRRL B-33077 | NK | NK | A | Lm3.1 | ST261 | III |
| NRRL B-33092 | NK | NK | A | Lm3.2 | ST262 | III |
| NRRL B-33181 | NK | NK | A | Lm3.5 | ST264 | III |
| NRRL B-33183 | NK | NK | A | Lm3.7 | ST265 | III |
| NRRL B-33185 | NK | NK | A | Lm3.9 | ST266 | III |
| NRRL B-33187 | NK | NK | A | Lm3.10 | ST267 | III |
| NRRL B-33190 | NK | NK | A | Lm3.12 | ST268 | III |
| NRRL B-33191 | NK | NK | A | Lm3.13 | ST269 | III |
| NRRL B-33105 | NK | NK | A | Lm3.3 | ST363 | III |
| NRRL B-33408 | 1997 | NK | A | Lm4.3 | ST563 | IV |
| OLM 8 | 1933 | USA | A | Lm3.42 | ST1214 | III |
| OLM 44 | 1950 | USA | A | 1.70_4b | ST1258 | I |
| OLM 37 | 1937 | USA | A | Lm3.52 | ST1263 | III |
| OLM 141 | 1956 | Romania | E | 1.13_4b_Sw87_EC1 | CC1 | I |
| 2934 | 2004 | NC, USA | E | 1.13_4b_Sw87_EC1 | CC1 | I |
| 2988 | 2005 | NC, USA | E | 1.13_4b_Sw87_EC1 | CC1 | I |
| 2990 | 2005 | NC, USA | E | 1.13_4b_Sw87_EC1 | CC1 | I |
| RM15644 | 2011 | CA, USA | E | 1.13_4b_Sw87_EC1 | CC1 | I |
| RM15655 | 2012 | CA, USA | E | 1.13_4b_Sw87_EC1 | CC1 | I |
| RM15683 | 2012 | CA, USA | E | 1.13_4b_Sw87_EC1 | CC1 | I |
| RM15691 | 2012 | CA, USA | E | 1.13_4b_Sw87_EC1 | CC1 | I |
| RM16551 | 2012 | CA, USA | E | 1.13_4b_Sw87_EC1 | CC1 | I |
| RM16550 | 2012 | CA, USA | E | 1.7_4b | CC4 | I |
| RM16554 | 2012 | CA, USA | E | 1.7_4b | CC4 | I |
| RM15654 | 2011 | CA, USA | E | 1.8_4b_US98_US02_EC2 | CC6 | I |
| RM16558 | 2012 | CA, USA | E | 1.8_4b_US98_US02_EC2 | CC6 | I |
| RM15647 | 2011 | CA, USA | E | 1.6_4b | CC217 | I |
| RM15685 | 2012 | CA, USA | E | 1.6_4b | CC217 | I |
| RM16547 | 2012 | CA, USA | E | 1.6_4b | CC217 | I |
| RM16559 | 2012 | CA, USA | E | 1.6_4b | CC217 | I |
| RM15666 | 2012 | CA, USA | E | 1.17_4b | ST382 | I |
| RM15668 | 2012 | CA, USA | E | 1.17_4b | ST382 | I |
| RM16540 | 2012 | CA, USA | E | 1.17_4b | ST382 | I |
| RM16541 | 2012 | CA, USA | E | 1.17_4b | ST382 | I |
| RM16542 | 2012 | CA, USA | E | 1.17_4b | ST382 | I |
| RM16544 | 2012 | CA, USA | E | 1.17_4b | ST382 | I |
| RM16553 | 2012 | CA, USA | E | 1.17_4b | ST382 | I |
| RM16556 | 2012 | CA, USA | E | 1.17_4b | ST382 | I |
| RM15653 | 2011 | CA, USA | E | 1.59_4b | ST639 | I |
| RM15657 | 2012 | CA, USA | E | 1.59_4b | ST639 | I |
| RM15673 | 2012 | CA, USA | E | 1.59_4b | ST639 | I |
| RM15677 | 2012 | CA, USA | E | 1.59_4b | ST639 | I |
| RM15697 | 2012 | CA, USA | E | 1.59_4b | ST639 | I |
| RM16545 | 2012 | CA, USA | E | 1.59_4b | ST639 | I |
| RM16546 | 2012 | CA, USA | E | 1.59_4b | ST639 | I |
| RM16548 | 2012 | CA, USA | E | 1.59_4b | ST639 | I |
| RM16549 | 2012 | CA, USA | E | 1.59_4b | ST639 | I |
| RM16552 | 2012 | CA, USA | E | 1.59_4b | ST639 | I |
| RM16555 | 2012 | CA, USA | E | 1.59_4b | ST639 | I |
| G3990 | 1985 | CA, USA | F | 1.11_4b_CA85_EC1 | CC1 | I |
| FDA 5 | 1986 | USA | F | 1.13_4b_Sw87_EC1 | CC1 | I |
| FDA 93 | 1986 | USA | F | 1.13_4b_Sw87_EC1 | CC1 | I |
| FDA 97 | 1986 | USA | F | 1.13_4b_Sw87_EC1 | CC1 | I |
| AT-16 | 1988 | USA | F | 1.13_4b_Sw87_EC1 | CC1 | I |
| F6812 | 1988 | USA | F | 1.13_4b_Sw87_EC1 | CC1 | I |
| FDA 34 | 1988 | USA | F | 1.13_4b_Sw87_EC1 | CC1 | I |
| FDA 96 | 1988 | USA | F | 1.13_4b_Sw87_EC1 | CC1 | I |
| LW-A1 | 2000 | USA | F | 1.13_4b_Sw87_EC1 | CC1 | I |
| LW-A4 | 2001 | USA | F | 1.13_4b_Sw87_EC1 | CC1 | I |
| LW-A45 | 2001 | USA | F | 1.13_4b_Sw87_EC1 | CC1 | I |
| LW-A46 | 2001 | USA | F | 1.13_4b_Sw87_EC1 | CC1 | I |
| LW-A6 | 2001 | USA | F | 1.13_4b_Sw87_EC1 | CC1 | I |
| LW-A69 | 2001 | USA | F | 1.13_4b_Sw87_EC1 | CC1 | I |
| L0228 | 2003 | VA, USA | F | 1.13_4b_Sw87_EC1 | CC1 | I |
| L0328 | 2003 | VA, USA | F | 1.13_4b_Sw87_EC1 | CC1 | I |
| LW-A32 | 2003 | USA | F | 1.13_4b_Sw87_EC1 | CC1 | I |
| BS-26 | 2003 | USA | F | 1.16_4b | CC1 | I |
| L0616 | 2004 | VA, USA | F | 1.13_4b_Sw87_EC1 | CC1 | I |
| L0617 | 2004 | VA, USA | F | 1.13_4b_Sw87_EC1 | CC1 | I |
| FDA 100 | 1986 | USA | F | 1.2_4b_UK88_EC1a | CC2 | I |
| FDA 11 | 1987 | USA | F | 1.4_4b_EC1a | CC2 | I |
| FDA 35 | 1988 | USA | F | 1.2_4b_UK88_EC1a | CC2 | I |
| F7194 | 1989 | USA | F | 1.2_4b_UK88_EC1a | CC2 | I |
| F7635 | 1989 | USA | F | 1.2_4b_UK88_EC1a | CC2 | I |
| F7666 | 1989 | USA | F | 1.2_4b_UK88_EC1a | CC2 | I |
| F7870 | 1989 | USA | F | 1.2_4b_UK88_EC1a | CC2 | I |
| FDA 10 | 1998 | USA | F | 1.2_4b_UK88_EC1a | CC2 | I |
| 82a-2 | 2003 | NC, USA | F | 1.2_4b_UK88_EC1a | CC2 | I |
| LW-A84 | 2004 | USA | F | 1.2_4b_UK88_EC1a | CC2 | I |
| LW-A85 | 2004 | USA | F | 1.2_4b_UK88_EC1a | CC2 | I |
| LW-A87 | 2004 | USA | F | 1.2_4b_UK88_EC1a | CC2 | I |
| LW-A88 | 2004 | USA | F | 1.2_4b_UK88_EC1a | CC2 | I |
| LW-A89 | 2004 | USA | F | 1.2_4b_UK88_EC1a | CC2 | I |
| LW-A90 | 2004 | USA | F | 1.2_4b_UK88_EC1a | CC2 | I |
| LW-A91 | 2004 | USA | F | 1.2_4b_UK88_EC1a | CC2 | I |
| LW-A92 | 2004 | USA | F | 1.2_4b_UK88_EC1a | CC2 | I |
| LW-A100 | 2005 | USA | F | 1.2_4b_UK88_EC1a | CC2 | I |
| LW-A101 | 2005 | USA | F | 1.2_4b_UK88_EC1a | CC2 | I |
| LW-A102 | 2005 | USA | F | 1.2_4b_UK88_EC1a | CC2 | I |
| LW-A103 | 2005 | USA | F | 1.2_4b_UK88_EC1a | CC2 | I |
| LW-A104 | 2005 | USA | F | 1.2_4b_UK88_EC1a | CC2 | I |
| LW-A98 | 2005 | USA | F | 1.2_4b_UK88_EC1a | CC2 | I |
| LW-A99 | 2005 | USA | F | 1.2_4b_UK88_EC1a | CC2 | I |
| GA 311 | 2005 | GA, USA | F | 1.1_4b_MA83_EC1a | CC2 | I |
| LW-A112 | 2006 | USA | F | 1.2_4b_UK88_EC1a | CC2 | I |
| LW-A130 | 2006 | USA | F | 1.2_4b_UK88_EC1a | CC2 | I |
| 1343 | 1992 | USA | F | 1.8_4b_US98_US02_EC2 | CC6 | I |
| 34-2b | 2003 | NC, USA | F | 1.8_4b_US98_US02_EC2 | CC6 | I |
| 1117 | 2003 | NC, USA | F | 1.8_4b_US98_US02_EC2 | CC6 | I |
| L0226 | 2003 | VA, USA | F | 1.8_4b_US98_US02_EC2 | CC6 | I |
| L0315 | 2003 | VA, USA | F | 1.8_4b_US98_US02_EC2 | CC6 | I |
| L0327 | 2003 | VA, USA | F | 1.8_4b_US98_US02_EC2 | CC6 | I |
| LW-A61 | 2003 | USA | F | 1.8_4b_US98_US02_EC2 | CC6 | I |
| L0603 | 2004 | VA, USA | F | 1.8_4b_US98_US02_EC2 | CC6 | I |
| L0623 | 2004 | VA, USA | F | 1.8_4b_US98_US02_EC2 | CC6 | I |
| L0704 | 2004 | VA, USA | F | 1.8_4b_US98_US02_EC2 | CC6 | I |
| L0719 | 2004 | VA, USA | F | 1.8_4b_US98_US02_EC2 | CC6 | I |
| L0720 | 2004 | VA, USA | F | 1.8_4b_US98_US02_EC2 | CC6 | I |
| L0724 | 2004 | VA, USA | F | 1.8_4b_US98_US02_EC2 | CC6 | I |
| L0928 | 2004 | VA, USA | F | 1.8_4b_US98_US02_EC2 | CC6 | I |
| L0929 | 2004 | VA, USA | F | 1.8_4b_US98_US02_EC2 | CC6 | I |
| L0930 | 2004 | VA, USA | F | 1.8_4b_US98_US02_EC2 | CC6 | I |
| LW-A105 | 2004 | USA | F | 1.8_4b_US98_US02_EC2 | CC6 | I |
| LW-A86 | 2004 | USA | F | 1.8_4b_US98_US02_EC2 | CC6 | I |
| 2688 | 2005 | MI, USA | F | 1.8_4b_US98_US02_EC2 | CC6 | I |
| LW-A109 | 2006 | USA | F | 1.77_4b | CC218 | I |
| FDA 107 | 1988 | USA | F | 1.63_4b | CC315 | I |
| F8027 | 2011 | USA | F | 1.63_4b | CC315 | I |
| 2509 | 2005 | MI, USA | F | 1.45_4b | CC388 | I |
| LW-A13 | 2001 | USA | F | 1.46_4b | CC554 | I |
| 18-2a | 2003 | NC, USA | F | 1.60_4b | CC554 | I |
| 128b-1 | 2004 | NC, USA | F | 1.60_4b | CC554 | I |
| 363b-1 | 2005 | NC, USA | F | 1.60_4b | CC554 | I |
| 491a-5 | 2006 | NC, USA | F | 1.60_4b | CC554 | I |
| LW-A125 | 2006 | USA | F | 1.59_4b | ST639 | I |
| NRRL B-33426 | NK | NK | F | Lm3.41 | ST1203 | III |
| OLM 10 | 1933 | USA | H | 1.13_4b_Sw87_EC1 | CC1 | I |
| OLM 15 | 1934 | USA | H | 1.16_4b | CC1 | I |
| OLM 18 | 1934 | USA | H | 1.16_4b | CC1 | I |
| OLM 61 | 1951 | Ontario, Canada | H | 1.13_4b_Sw87_EC1 | CC1 | I |
| OLM 71 | 1953 | Ontario, Canada | H | 1.13_4b_Sw87_EC1 | CC1 | I |
| OLM 74 | 1953 | Ontario, Canada | H | 1.13_4b_Sw87_EC1 | CC1 | I |
| OLM 93 | 1954 | Canada | H | 1.16_4b | CC1 | I |
| OLM 97 | 1954 | Nova Scotia, Canada | H | 1.13_4b_Sw87_EC1 | CC1 | I |
| OLM 98 | 1955 | Nova Scotia, Canada | H | 1.13_4b_Sw87_EC1 | CC1 | I |
| OLM 125 | 1959 | Ontario, Canada | H | 1.13_4b_Sw87_EC1 | CC1 | I |
| OLM 142 | 1960 | Newfoundland, Canada | H | 1.13_4b_Sw87_EC1 | CC1 | I |
| OLM 143 | 1961 | Ontario, Canada | H | 1.13_4b_Sw87_EC1 | CC1 | I |
| OLM 147 | 1961 | BC, Canada | H | 1.13_4b_Sw87_EC1 | CC1 | I |
| OLM 152 | 1963 | Newfoundland, Canada | H | 1.13_4b_Sw87_EC1 | CC1 | I |
| J2213 | 2003 | AZ, USA | H | 1.13_4b_Sw87_EC1 | CC1 | I |
| J2269 | 2003 | GA, USA | H | 1.13_4b_Sw87_EC1 | CC1 | I |
| J2275 | 2003 | PA, USA | H | 1.13_4b_Sw87_EC1 | CC1 | I |
| J2282 | 2003 | MD, USA | H | 1.13_4b_Sw87_EC1 | CC1 | I |
| J2288 | 2003 | TX, USA | H | 1.13_4b_Sw87_EC1 | CC1 | I |
| J2302 | 2003 | CA, USA | H | 1.13_4b_Sw87_EC1 | CC1 | I |
| J2313 | 2003 | TX, USA | H | 1.13_4b_Sw87_EC1 | CC1 | I |
| J2327 | 2003 | MI, USA | H | 1.13_4b_Sw87_EC1 | CC1 | I |
| J2353 | 2003 | IL, USA | H | 1.13_4b_Sw87_EC1 | CC1 | I |
| J2391 | 2003 | TX, USA | H | 1.13_4b_Sw87_EC1 | CC1 | I |
| J2584 | 2003 | VT, USA | H | 1.13_4b_Sw87_EC1 | CC1 | I |
| J2854 | 2004 | AZ, USA | H | 1.13_4b_Sw87_EC1 | CC1 | I |
| J2985 | 2004 | IL, USA | H | 1.13_4b_Sw87_EC1 | CC1 | I |
| J3082 | 2004 | GA, USA | H | 1.13_4b_Sw87_EC1 | CC1 | I |
| J3106 | 2004 | NY, USA | H | 1.13_4b_Sw87_EC1 | CC1 | I |
| J3133 | 2004 | TX, USA | H | 1.13_4b_Sw87_EC1 | CC1 | I |
| J3180 | 2004 | CO, USA | H | 1.13_4b_Sw87_EC1 | CC1 | I |
| J3232 | 2004 | OK, USA | H | 1.13_4b_Sw87_EC1 | CC1 | I |
| J3410 | 2005 | SC, USA | H | 1.13_4b_Sw87_EC1 | CC1 | I |
| J3422 | 2005 | LA, USA | H | 1.16_4b | CC1 | I |
| J3535 | 2005 | RI, USA | H | 1.13_4b_Sw87_EC1 | CC1 | I |
| J3559 | 2005 | GA, USA | H | 1.13_4b_Sw87_EC1 | CC1 | I |
| J3592 | 2005 | ME, USA | H | 1.13_4b_Sw87_EC1 | CC1 | I |
| J3709 | 2005 | NH, USA | H | 1.13_4b_Sw87_EC1 | CC1 | I |
| J3799 | 2005 | CT, USA | H | 1.13_4b_Sw87_EC1 | CC1 | I |
| J3916 | 2006 | NM, USA | H | 1.13_4b_Sw87_EC1 | CC1 | I |
| J4001 | 2006 | TX, USA | H | 1.13_4b_Sw87_EC1 | CC1 | I |
| J4099 | 2006 | VA, USA | H | 1.13_4b_Sw87_EC1 | CC1 | I |
| J4116 | 2006 | ME, USA | H | 1.13_4b_Sw87_EC1 | CC1 | I |
| J4187 | 2006 | WI, USA | H | 1.13_4b_Sw87_EC1 | CC1 | I |
| J4253 | 2006 | TN, USA | H | 1.13_4b_Sw87_EC1 | CC1 | I |
| J4274 | 2006 | NH, USA | H | 1.13_4b_Sw87_EC1 | CC1 | I |
| J4297 | 2006 | PA, USA | H | 1.13_4b_Sw87_EC1 | CC1 | I |
| J4316 | 2006 | IA, USA | H | 1.13_4b_Sw87_EC1 | CC1 | I |
| J4429 | 2007 | OR, USA | H | 1.13_4b_Sw87_EC1 | CC1 | I |
| J4600 | 2007 | OK, USA | H | 1.13_4b_Sw87_EC1 | CC1 | I |
| J4685 | 2007 | MO, USA | H | 1.13_4b_Sw87_EC1 | CC1 | I |
| 2008-114 | 2008 | NC, USA | H | 1.13_4b_Sw87_EC1 | CC1 | I |
| 2008-451 | 2008 | NC, USA | H | 1.13_4b_Sw87_EC1 | CC1 | I |
| J4950 | 2008 | WI, USA | H | 1.13_4b_Sw87_EC1 | CC1 | I |
| J4977 | 2008 | NC, USA | H | 1.13_4b_Sw87_EC1 | CC1 | I |
| J4979 | 2008 | TX, USA | H | 1.13_4b_Sw87_EC1 | CC1 | I |
| J5043 | 2008 | MA, USA | H | 1.13_4b_Sw87_EC1 | CC1 | I |
| J5080 | 2008 | NM, USA | H | 1.13_4b_Sw87_EC1 | CC1 | I |
| J5095 | 2008 | MD, USA | H | 1.13_4b_Sw87_EC1 | CC1 | I |
| J5136 | 2008 | SC, USA | H | 1.13_4b_Sw87_EC1 | CC1 | I |
| J5202 | 2008 | MS, USA | H | 1.13_4b_Sw87_EC1 | CC1 | I |
| J5354 | 2008 | UT, USA | H | 1.13_4b_Sw87_EC1 | CC1 | I |
| J5392 | 2008 | KY, USA | H | 1.13_4b_Sw87_EC1 | CC1 | I |
| J5478 | 2008 | IL, USA | H | 1.13_4b_Sw87_EC1 | CC1 | I |
| 2010-0055 | 2010 | NC, USA | H | 1.13_4b_Sw87_EC1 | CC1 | I |
| 2010-0072A | 2010 | NC, USA | H | 1.13_4b_Sw87_EC1 | CC1 | I |
| OLM 11 | 1933 | USA | H | 1.2_4b_UK88_EC1a | CC2 | I |
| OLM 77 | 1954 | Ontario, Canada | H | 1.2_4b_UK88_EC1a | CC2 | I |
| OLM 78 | 1954 | Ontario, Canada | H | 1.2_4b_UK88_EC1a | CC2 | I |
| OLM 102 | 1955 | Nova Scotia, Canada | H | 1.2_4b_UK88_EC1a | CC2 | I |
| OLM 117 | 1956 | Nova Scotia, Canada | H | 1.2_4b_UK88_EC1a | CC2 | I |
| OLM 118 | 1956 | Nova Scotia, Canada | H | 1.2_4b_UK88_EC1a | CC2 | I |
| OLM 120 | 1957 | Canada | H | 1.2_4b_UK88_EC1a | CC2 | I |
| OLM 121 | 1957 | Ontario, Canada | H | 1.2_4b_UK88_EC1a | CC2 | I |
| OLM 124 | 1958 | Ontario, Canada | H | 1.2_4b_UK88_EC1a | CC2 | I |
| OLM 138 | 1961 | Ontario, Canada | H | 1.2_4b_UK88_EC1a | CC2 | I |
| OLM 144 | 1961 | Brazil | H | 1.2_4b_UK88_EC1a | CC2 | I |
| 4b1 | 1962 | Germany | H | 1.2_4b_UK88_EC1a | CC2 | I |
| OLM 151 | 1963 | Newfoundland, Canada | H | 1.2_4b_UK88_EC1a | CC2 | I |
| OLM 153 | 1963 | Newfoundland, Canada | H | 1.2_4b_UK88_EC1a | CC2 | I |
| OLM 157 | 1964 | Ontario, Canada | H | 1.2_4b_UK88_EC1a | CC2 | I |
| B068 | 1983 | MA, USA | H | 1.1_4b_MA83_EC1a | CC2 | I |
| J2967 | 2004 | CA, USA | H | 1.2_4b_UK88_EC1a | CC2 | I |
| J3290 | 2004 | ME, USA | H | 1.2_4b_UK88_EC1a | CC2 | I |
| J3419 | 2005 | CA, USA | H | 1.2_4b_UK88_EC1a | CC2 | I |
| J3768 | 2005 | CO, USA | H | 1.2_4b_UK88_EC1a | CC2 | I |
| J3921 | 2006 | CT, USA | H | 1.2_4b_UK88_EC1a | CC2 | I |
| 2007-582 | 2007 | NC, USA | H | 1.2_4b_UK88_EC1a | CC2 | I |
| J4503 | 2007 | NYC, NY, USA | H | 1.2_4b_UK88_EC1a | CC2 | I |
| J4559 | 2007 | MA, USA | H | 1.2_4b_UK88_EC1a | CC2 | I |
| J4811 | 2007 | MA, USA | H | 1.2_4b_UK88_EC1a | CC2 | I |
| J4948 | 2008 | GA, USA | H | 1.2_4b_UK88_EC1a | CC2 | I |
| J4954 | 2008 | CT, USA | H | 1.2_4b_UK88_EC1a | CC2 | I |
| J5074 | 2008 | WI, USA | H | 1.2_4b_UK88_EC1a | CC2 | I |
| J5375 | 2008 | WA, USA | H | 1.2_4b_UK88_EC1a | CC2 | I |
| OLM 12 | 1934 | CT, USA | H | 1.7_4b | CC4 | I |
| OLM 30 | 1938 | Uruguay | H | 1.7_4b | CC4 | I |
| OLM 36 | 1940 | Argentina | H | 1.7_4b | CC4 | I |
| J2422 | 2003 | RI, USA | H | 1.7_4b | CC4 | I |
| J3070 | 2004 | TN, USA | H | 1.62_4b | CC4 | I |
| J3120 | 2004 | WA, USA | H | 1.7_4b | CC4 | I |
| J3309 | 2005 | IA, USA | H | 1.7_4b | CC4 | I |
| J3415 | 2005 | TX, USA | H | 1.7_4b | CC4 | I |
| J3554 | 2005 | MS, USA | H | 1.62_4b | CC4 | I |
| J3618 | 2005 | NJ, USA | H | 1.7_4b | CC4 | I |
| J3684 | 2005 | OR, USA | H | 1.7_4b | CC4 | I |
| J3762 | 2005 | IN, USA | H | 1.62_4b | CC4 | I |
| J3795 | 2005 | AZ, USA | H | 1.7_4b | CC4 | I |
| J3909 | 2006 | OR, USA | H | 1.7_4b | CC4 | I |
| J4428 | 2007 | OH, USA | H | 1.62_4b | CC4 | I |
| J4434 | 2007 | TN, USA | H | 1.7_4b | CC4 | I |
| J4435 | 2007 | WI, USA | H | 1.62_4b | CC4 | I |
| J4465 | 2007 | CO, USA | H | 1.7_4b | CC4 | I |
| J5038 | 2008 | MI, USA | H | 1.62_4b | CC4 | I |
| H6383 | 1996 | OH, USA | H | 1.8_4b_US98_US02_EC2 | CC6 | I |
| H3396 | 1997 | CA, USA | H | 1.8_4b_US98_US02_EC2 | CC6 | I |
| 1450 | 1998 | USA | H | 1.9_4b_US98_EC2 | CC6 | I |
| 1452 | 1998 | USA | H | 1.8_4b_US98_US02_EC2 | CC6 | I |
| 1453 | 1998 | USA | H | 1.8_4b_US98_US02_EC2 | CC6 | I |
| 1463 | 2002 | USA | H | 1.8_4b_US98_US02_EC2 | CC6 | I |
| 1465 | 2002 | USA | H | 1.8_4b_US98_US02_EC2 | CC6 | I |
| J2206 | 2003 | NJ, USA | H | 1.8_4b_US98_US02_EC2 | CC6 | I |
| J2230 | 2003 | MA, USA | H | 1.9_4b_US98_EC2 | CC6 | I |
| J2446 | 2003 | OH, USA | H | 1.9_4b_US98_EC2 | CC6 | I |
| NRRL B-33589 | 2003 | MD, USA | H | 1.8_4b_US98_US02_EC2 | CC6 | I |
| NRRL B-33591 | 2003 | GA, USA | H | 1.8_4b_US98_US02_EC2 | CC6 | I |
| J2685 | 2004 | NY, USA | H | 1.8_4b_US98_US02_EC2 | CC6 | I |
| J3006 | 2004 | TX, USA | H | 1.8_4b_US98_US02_EC2 | CC6 | I |
| J3033 | 2004 | IL, USA | H | 1.8_4b_US98_US02_EC2 | CC6 | I |
| J3075 | 2004 | CT, USA | H | 1.8_4b_US98_US02_EC2 | CC6 | I |
| J3170 | 2004 | MI, USA | H | 1.8_4b_US98_US02_EC2 | CC6 | I |
| J3200 | 2004 | CT, USA | H | 1.8_4b_US98_US02_EC2 | CC6 | I |
| J3333 | 2004 | MN, USA | H | 1.8_4b_US98_US02_EC2 | CC6 | I |
| J3527 | 2005 | WI, USA | H | 1.8_4b_US98_US02_EC2 | CC6 | I |
| J3565 | 2005 | IL, USA | H | 1.8_4b_US98_US02_EC2 | CC6 | I |
| J3606 | 2005 | MI, USA | H | 1.8_4b_US98_US02_EC2 | CC6 | I |
| J3785 | 2005 | MA, USA | H | 1.9_4b_US98_EC2 | CC6 | I |
| J3840 | 2005 | MD, USA | H | 1.8_4b_US98_US02_EC2 | CC6 | I |
| J3881 | 2006 | CO, USA | H | 1.8_4b_US98_US02_EC2 | CC6 | I |
| J4101 | 2006 | WV, USA | H | 1.8_4b_US98_US02_EC2 | CC6 | I |
| J4105 | 2006 | AL, USA | H | 1.8_4b_US98_US02_EC2 | CC6 | I |
| J4120 | 2006 | OH, USA | H | 1.8_4b_US98_US02_EC2 | CC6 | I |
| J4210 | 2006 | GA, USA | H | 1.8_4b_US98_US02_EC2 | CC6 | I |
| J4485 | 2007 | MA, USA | H | 1.8_4b_US98_US02_EC2 | CC6 | I |
| J4610 | 2007 | ME, USA | H | 1.8_4b_US98_US02_EC2 | CC6 | I |
| J5172 | 2008 | MN, USA | H | 1.8_4b_US98_US02_EC2 | CC6 | I |
| 2009-0079 | 2009 | NC, USA | H | 1.8_4b_US98_US02_EC2 | CC6 | I |
| 2009-0117 | 2009 | NC, USA | H | 1.8_4b_US98_US02_EC2 | CC6 | I |
| J3976 | 2006 | MA, USA | H | 1.6_4b | CC217 | I |
| J4179 | 2006 | RI, USA | H | 1.6_4b | CC217 | I |
| J4726 | 2007 | FL, USA | H | 1.6_4b | CC217 | I |
| J5032 | 2008 | PA, USA | H | 1.6_4b | CC217 | I |
| J2255 | 2003 | GA, USA | H | 1.17_4b | ST382 | I |
| J3139 | 2004 | WI, USA | H | 1.17_4b | ST382 | I |
| J3913 | 2006 | IN, USA | H | 1.17_4b | ST382 | I |
| J4016 | 2006 | SC, USA | H | 1.17_4b | ST382 | I |
| 2007-0904 | 2007 | NC, USA | H | 1.17_4b | ST382 | I |
| J4500 | 2007 | NE, USA | H | 1.17_4b | ST382 | I |
| J5000 | 2008 | VA, USA | H | 1.17_4b | ST382 | I |
| J3085 | 2004 | MD, USA | H | 1.45_4b | CC388 | I |
| J3115 | 2004 | VA, USA | H | 1.73_4b | CC388 | I |
| J3728 | 2005 | NY, USA | H | 1.45_4b | CC388 | I |
| J3793 | 2005 | WV, USA | H | 1.45_4b | CC388 | I |
| J4291 | 2006 | NY, USA | H | 1.76_4b | CC388 | I |
| J4548 | 2007 | WI, USA | H | 1.45_4b | CC388 | I |
| J4713 | 2007 | RI, USA | H | 1.45_4b | CC388 | I |
| J5502 | 2008 | NM, USA | H | 1.45_4b | CC388 | I |
| J4460 | 2007 | GA, USA | H | ND^3^ | CC389 | I |
| J4875 | 2007 | NY, USA | H | ND^3^ | CC389 | I |
| 2001-7R | 2001 | NC, USA | H | 1.60_4b | CC554 | I |
| 2001-8R | 2001 | NC, USA | H | 1.60_4b | CC554 | I |
| J3026 | 2004 | CT, USA | H | 1.46_4b | CC554 | I |
| J3053 | 2004 | MI, USA | H | 1.46_4b | CC554 | I |
| J3195 | 2004 | OH, USA | H | 1.46_4b | CC554 | I |
| 2006-296 | 2006 | NC, USA | H | 1.60_4b | CC554 | I |
| 2007-454 | 2007 | NC, USA | H | 1.60_4b | CC554 | I |
| 2007-618 | 2007 | NC, USA | H | 1.46_4b | CC554 | I |
| J4458 | 2007 | MN, USA | H | 1.46_4b | CC554 | I |
| J4490 | 2007 | SC, USA | H | 1.46_4b | CC554 | I |
| 2008-894 | 2008 | NC, USA | H | 1.60_4b | CC554 | I |
| J4953 | 2008 | OH, USA | H | 1.46_4b | CC554 | I |
| WS1 | 2000 | NC, USA | H | 1.42_4b_NC00 | ST558 | I |
| J3215 | 2004 | NJ, USA | H | 1.42_4b_NC00 | ST558 | I |
| J4045 | 2006 | MO, USA | H | 1.42_4b_NC00 | ST558 | I |
| 2007-583 | 2007 | NC, USA | H | 1.42_4b_NC00 | ST558 | I |
| J3155 | 2004 | IL, USA | H | 1.59_4b | ST639 | I |
| J4696 | 2007 | SD, USA | H | 1.59_4b | ST639 | I |
| J5421 | 2008 | FL, USA | H | 1.59_4b | ST639 | I |
| J2621 | 2003 | OR, USA | H | 1.5_4b | ST663 | I |
| J3917 | 2006 | CA, USA | H | 1.5_4b | ST663 | I |
| J4010 | 2006 | NE, USA | H | 1.5_4b | ST663 | I |
| J4109 | 2006 | NJ, USA | H | 1.5_4b | ST663 | I |
| J3751 | 2005 | OH, USA | H | 1.58_4b | ST666 | I |
| J4432 | 2007 | PA, USA | H | 1.58_4b | ST666 | I |
| J3182 | 2004 | OR, USA | H | 1.71_4b | ST688 | I |
| 2007-584 | 2007 | NC, USA | H | 1.74_4b | ST1039 | I |
| 2013L-5086 | 2013 | NY, USA | H | ND^3^ | ST1039 | I |
| 2013L-5144 | 2013 | NY, USA | H | ND^3^ | ST1039 | I |
| 2014L-6500 | 2014 | NC, USA | H | ND^3^ | ST1039 | I |
| 2014L-6552 | 2014 | NC, USA | H | ND^3^ | ST1039 | I |
| J5166 | 2008 | KS, USA | H | 1.64_4b | ST1061 | I |
| J2479 | 2003 | MI, USA | H | Lm3.42 | ST1214 | III |
| J2571 | 2003 | KY, USA | H | Lm3.42 | ST1214 | III |
| J3720 | 2005 | PA, USA | H | Lm3.42 | ST1214 | III |
| J4221 | 2006 | MD, USA | H | 1.75_4b | ST1256 | I |
| OLM 80 | 1954 | Canada | H | 1.70_4b | ST1258 | I |
| OLM 115 | 1955 | Canada | H | 1.70_4b | ST1258 | I |
| OLM 116 | 1956 | Canada | H | 1.70_4b | ST1258 | I |
| (HI) 267 | 1999 | HI, USA | H | Lm3.49 | ST1264 | III |

**Table S1.**

List of serotype 4b *Listeria monocytogenes* isolates investigated in this study.

^1^ Numbers of isolates from different sources: H, Human clinical isolates; F, isolates from food or food processing environments; A, isolates from non-human animals; E, isolates from the natural environment With the exception of strain OLM 141 (mud) and 2934, 2988 and 2990 which were from water treatment plant effluent, all other isolates from the natural environment were from California watershed surveys (1)

^2^ NK, Not known

^3^ ND, not determined

^4^ Lineage was determined by MLGT, as described (2)

**REFERENCES**

1. Cooley MB, Quiñones B, Oryang D, Mandrell RE, Gorski L. 2014. Prevalence of shiga toxin producing *Escherichia coli*, *Salmonella enterica*, and *Listeria monocytogenes* at public access watershed sites in a California Central Coast agricultural region. Front Cell Infect Microbiol. 4:30. doi: 10.3389/fcimb.2014.00030. eCollection 2014. Erratum in: Front Cell Infect Microbiol. 2014;4:58.

Ward TJ, Usgaard T, Evans P. 2010. A targeted multilocus genotyping assay for lineage, serogroup, and epidemic clone typing of *Listeria monocytogenes*. Appl Environ Microbiol. 76(19):6680-4. doi: 10.1128/AEM.01008-10. Epub 2010 Aug 13.
