# Supplementary material for: Listeria monocytogenes Source Distribution Analysis Indicates Regional Heterogeneity and Ecological Niche Preference among Serotype 4b Clones
Source: mBio. 2018 Apr 17;9(2):e00396-18. doi: 10.1128/mBio.00396-18 (PMC5904418; doi:10.1128/mBio.00396-18)
Supplement: TABLE S2 [file mbo002183818st2.docx]

| **Table S2. Source distribution of serotype 4b clones** | | | | | | | | | | | | |
| --- | --- | --- | --- | --- | --- | --- | --- | --- | --- | --- | --- | --- |
|  | **No. of isolates** | | | | | **Statistical significance of differences in distribution** | | | | | | |
|  | **No. (% isolates)** | **Source^1^** | | | | **Across Sources** | **Pairwise comparisons** | | | | | |
|  |  | **H** | **F** | **E** | **A** |  | **F vs. H** | **E vs. H** | **E vs. F** | **A vs. H** | **A vs. F** | **A vs. E** |
| **Total** | 347 | 209 | 77 | 36 | 25 |  |  |  |  |  |  |  |
| **LINEAGE I Total** | 329 (94.8%) | 205 | 76 | 36 | 12 |  |  |  |  |  |  |  |
|  |  |  |  |  |  |  |  |  |  |  |  |  |
| **LIN. I**  **Clone** |  |  |  |  |  |  |  |  |  |  |  |  |
| CC1 | 104 (30%) | 66 | 20 | 9 | 9 | - | - | - | - | - | - | - |
| CC2 | 57 (16.4%) | 29 | 27 | 0 | 1 | **** | *** | * | **** | - | ** | - |
| CC6 | 55 (15.9%) | 34 | 19 | 2 | 0 | - | - | - | * | * | ** | - |
| CC4 | 22 (6.3%) | 19 | 0 | 2 | 1 | - | ** | - | - | - | - | - |
| CC554 | 17 (4.9%) | 12 | 5 | 0 | 0 | - | - | - | - | - | - | - |
| ST382 | 15 (4.3%) | 7 | 0 | 8 | 0 | **** | - | *** | **** | - | - | * |
| ST639 | 15 (4.3%) | 3 | 1 | 11 | 0 | **** | - | **** | **** | - | - | ** |
| CC388 | 9 (2.6%) | 8 | 1 | 0 | 0 | - | - | - | - | - | - | - |
| CC217 | 8 (2.3%) | 4 | 0 | 4 | 0 | - | - | * | ** | - | - | - |
| ST1039 | 5 (1.4%) | 5 | 0 | 0 | 0 | - | - | - | - | - | - | - |
| ST558 | 4 (1.2%) | 4 | 0 | 0 | 0 | - | - | - | - | - | - | - |
| ST663 | 4 (1.2%) | 4 | 0 | 0 | 0 | - | - | - | - | - | - | - |
| ST1258 | 4 (1.2%) | 3 | 0 | 0 | 1 | - | - | - | - | - | - | - |
| CC315 | 2 (0.6%) | 0 | 2 | 0 | 0 | - | - | - | - | - | - | - |
| CC389 | 2 (0.6%) | 2 | 0 | 0 | 0 | - | - | - | - | - | - | - |
| ST666 | 2 (0.6%) | 2 | 0 | 0 | 0 | - | - | - | - | - | - | - |
| CC218 | 1 (0.3%) | 0 | 1 | 0 | 0 | - | - | - | - | - | - | - |
| ST688 | 1 (0.3%) | 1 | 0 | 0 | 0 | - | - | - | - | - | - | - |
| ST1061 | 1 (0.3%) | 1 | 0 | 0 | 0 | - | - | - | - | - | - | - |
| ST1256 | 1 (0.3%) | 1 | 0 | 0 | 0 | - | - | - | - | - | - | - |
|  | | | | | | | | | | | | |
| **LINEAGE III Total** | 17 (4.9%) | 4 | 1 | 0 | 12 | **** | - | - | - | **** | **** | **** |
| **LIN. III Clone** |  |  |  |  |  |  |  |  |  |  |  |  |
| ST1214 | 4 (1.2%) | 3 | 0 | 0 | 1 | - | - | - | - | - | - | - |
| ST261 | 2 (0.6%) | 0 | 0 | 0 | 2 | - | - | - | - | - | - | - |
| ST262 | 1 (0.3%) | 0 | 0 | 0 | 1 | - | - | - | - | - | - | - |
| ST264 | 1 (0.3%) | 1 | 0 | 0 | 0 | - | - | - | - | - | - | - |
| ST265 | 1 (0.3%) | 0 | 0 | 0 | 1 | - | - | - | - | - | - | - |
| ST266 | 1 (0.3%) | 0 | 0 | 0 | 1 | - | - | - | - | - | - | - |
| ST267 | 1 (0.3%) | 0 | 0 | 0 | 1 | - | - | - | - | - | - | - |
| ST268 | 1 (0.3%) | 0 | 0 | 0 | 1 | - | - | - | - | - | - | - |
| ST269 | 1 (0.3%) | 0 | 0 | 0 | 1 | - | - | - | - | - | - | - |
| ST363 | 1 (0.3%) | 0 | 0 | 0 | 1 | - | - | - | - | - | - | - |
| ST1203 | 1 (0.3%) | 0 | 1 | 0 | 0 | - | - | - | - | - | - | - |
| ST1263 | 1 (0.3%) | 0 | 0 | 0 | 1 | - | - | - | - | - | - | - |
| ST1264 | 1 (0.3%) | 1 | 0 | 0 | 0 | - | - | - | - | - | - | - |
|  | | | | | | | | | | | | |
| **LINEAGE IV Total** | 1 (0.3%) | 0 | 0 | 0 | 1 |  |  |  |  |  |  |  |
| **LIN. IV Clone** |  |  |  |  |  |  |  |  |  |  |  |  |
| ST563 | 1 (0.3%) | 0 | 0 | 0 | 1 | - | - | - | - | - | - | - |

**Table S2.**

Statistical analysis of the source distribution of serotype 4b *Listeria monocytogenes* clones.

^1^ Numbers of isolates from different sources: H, Human clinical isolates; F, isolates from food or food processing environments; A, isolates from non-human animals; E, isolates from the natural environment (all but one were from watersheds or water treatment plant effluent).

Significantly different source-associated distributions are indicated with *, **, ***, **** (P < 0.05, 0.01, 0.001, 0.0001, respectively).
